# Optimizing the hybridization chain reaction-fluorescence in situ hybridization (HCR-FISH) protocol for detection of microbes in sediments

**DOI:** 10.1007/s42995-021-00098-8

**Published:** 2021-04-30

**Authors:** Zeyu Jia, Yijing Dong, Heng Xu, Fengping Wang

**Affiliations:** 1 State Key Laboratory of Microbial Metabolism, Joint International Research Laboratory of Metabolic and Developmental Sciences, School of Life Sciences and Biotechnology, Shanghai Jiao Tong University, Shanghai, 200240 China; 2 School of Physics and Astronomy, Shanghai Jiao Tong University, Shanghai, 200240 China; 3 Institute of Natural Science, Shanghai Jiao Tong University, Shanghai, 200240 China; 4 School of Oceanography, Shanghai Jiao Tong University, Shanghai, 200240 China

**Keywords:** Fluorescence in situ hybridization, Hybridization chain reaction, HCR-FISH, Microbial detection, Sediment

## Abstract

Fluorescence in situ hybridization (FISH) is a canonical tool commonly used in environmental microbiology research to visualize targeted cells. However, the problems of low signal intensity and false-positive signals impede its widespread application. Alternatively, the signal intensity can be amplified by incorporating Hybridization Chain Reaction (HCR) with FISH, while the specificity can be improved through protocol modification and proper counterstaining. Here we optimized the HCR-FISH protocol for studying microbes in environmental samples, particularly marine sediments. Firstly, five sets of HCR initiator/amplifier pairs were tested on the laboratory-cultured bacterium *Escherichia coli* and the archaeon *Methanococcoides methylutens*, and two sets displayed high hybridization efficiency and specificity. Secondly, we tried to find the best combination of sample pretreatment methods and HCR-FISH protocol for environmental sample analysis with the aim of producing less false positive signals. Various detachment methods, extraction methods and formulas of hybridization buffer were tested using sediment samples. Thirdly, an image processing method was developed to enhance the DAPI signal of microbial cells against that of abiotic particles, providing a reliable reference for FISH imaging. In summary, our optimized HCR-FISH protocol showed promise to serve as an addendum to traditional FISH for research on environmental microbes.

**Supplementary Information:**

The online version contains supplementary material available at 10.1007/s42995-021-00098-8.

## Introduction

Fluorescence in situ hybridization (FISH) is a widely used research tool in studying the environmental microbial community (Yamaguchi and Kubota 2017). By labelling 16S rRNA, FISH can phylogenetically distinguish targeted microbes at the single-cell level. The applications of FISH in environmental research include quantification of specific microbial populations (Baptista et al. 2014; Buongiorno et al. 2017), cell-level exploration of spatial structure of microbial communities (Orcutt and Meile 2008; Wilen et al. 2008), and providing an indication of cell location for other high-resolution imaging techniques such as Nanoscale secondary ion mass spectrometry (nanoSIMS), Bio-Orthogonal Non-Canonical Amino acid Tagging (BONCAT), and Raman microscopy (Chen et al. 2014; Hatzenpichler et al. 2016; Huang et al. 2007).

In order to observe microbes with epi-fluorescence microscopes, the fluorescence signal of the targeted cells must be intensive and specific. High signal intensity ensures that targeted cells can be distinguished from background signals, and the high specificity ensures that untargeted cells and abiotic particles are not mis-recognized as targeted cells. The traditional FISH method usually works well on highly active microbes on both signal intensity and specificity (Wilen et al. 2008). However, microbial cells in sediment are typically less active and/or smaller in size than *E. coli* (Amann and Fuchs 2008; Orphan et al. 2009), with inadequate amounts of rRNA for traditional FISH to create signals intense enough to distinguish cells from the background (Fazi et al. 2007). In addition to using fluorophores with higher efficiency, a variety of signal amplification methods have been tried and combined with FISH to explore the microbial communities in sediments (Amann et al. 1995). One method is to design multiple probes targeting different loci of the same rRNA, thus strengthening the signal of each rRNA (Morris et al. 2002). Another method is to deploy a multi-labeled polynucleotide probe, e.g., using RNA transcripts from PCR amplicons of 16S and 23S rRNA genes, with multiple fluorescently labeled uridine incorporated during transcription (Pernthaler et al. 2002). Other methods have been borrowed from sensor technology, of which the catalyzed reporter deposition FISH (CARD-FISH) is the most well-reported (Kubota 2013). This method requires a DNA probe labeled with horseradish peroxidase (HRP). This enzyme can induce the reaction of hydroperoxide, fluorescence-labeled tyramide, and intracellular aromatic compounds, leading to a deposition of tyramide on the target site. Although this method significantly increases the fluorescence signal, some issues have restricted its application on sediment samples (Kubota 2013): the large molecular weight (~ 40 kDa) of HRP prevents the entrance of the probe into the cells, thus extra permeabilization should be undertaken prior to the hybridization; H_2_O_2_ is necessary for inactivation of intracellular peroxidase to avoid a false-positive result, however, this may degrade the nucleic acid (Massie et al. 1972).

Hybridization chain reaction (HCR) is another signal amplification method (Dirks and Pierce 2004). It is a strand displacement amplification method, where the nicked double-helix nucleotide strand is produced without the use of enzyme and a change of temperature. For example, in DNA HCR process, the presence of the initiator oligonucleotide triggers a repeating hybridization of two species of DNA hairpins. By combining the initiator with different type of molecules, such as aptamer (Bao et al. 2020; Jia et al. 2018; Zhang et al. 2020), antibodies (Choi et al. 2011), DNA probes (Yamaguchi et al. 2015b), nanoparticles (Zeng et al. 2019; Zhang et al. 2012), etc., the long-chain double-stranded DNA (dsDNA) can specifically locate the target molecule. Since the DNA hairpins can incorporate the fluorophore (Huang et al. 2011), nanoparticles (Gao et al. 2017; Miao et al. 2015; Wu et al. 2018), electrochemical indicator (Hou et al. 2015), etc., the amplified signal can be detected by diverse kinds of instruments, such as spectrophotometers, electrodes and transmission electron microscopes (Bi et al. 2017).

**Fig. 1:**
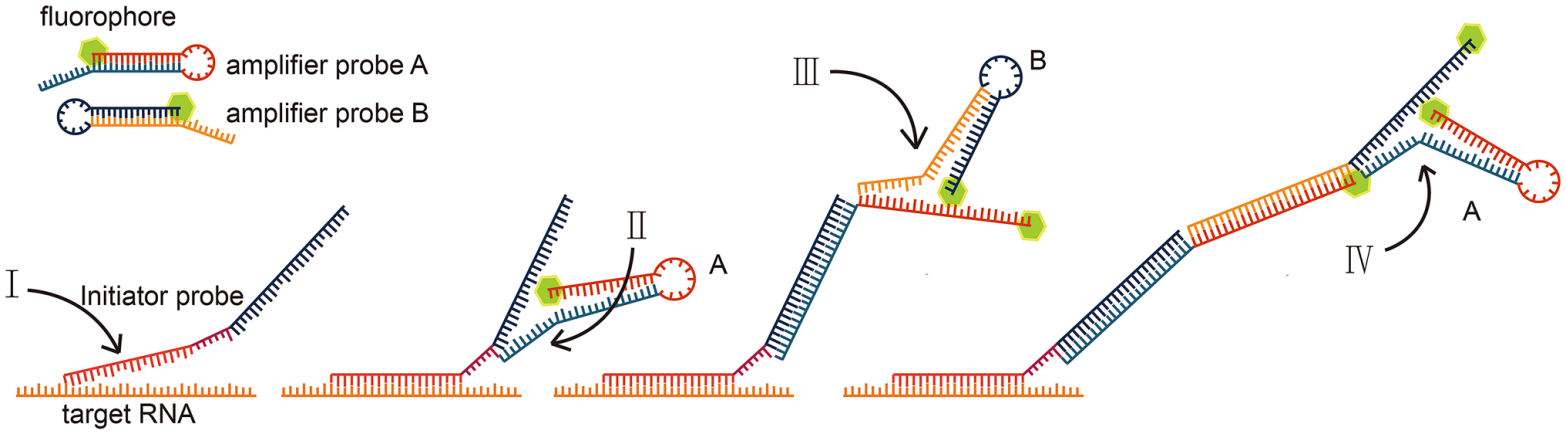
Mechanism of HCR-FISH. After the initiator probe hybridized to the target RNA (process I), amplifier probes could bind to the initiator step by step (process II, III, IV) (Dirks and Pierce 2004; Yamaguchi et al. 2015b)

Of the described methods, the combination of HCR with FISH for bio-imaging was first reported in 2010 (Choi et al. 2010). In HCR-FISH, the aforementioned initiators in the HCR system are concatenated to the probes of traditional FISH as initiator probes. Unlike the fluorescently labeled FISH probe, the initiator probes of HCR-FISH are not labeled. Two amplifier probes, A and B, are also required for HCR-FISH, each of which is fluorescently labeled and has a stable hairpin structure with free tails on their 5′ terminal. At the beginning, initiator probes hybridize with targeted intracellular RNA, leaving their initiator sequence unpaired (Fig. 1, process I). After the removal of excessive initiator probes, amplifier probes A and B can be added. The unpaired initiator sequence binds to the unpaired tail of probe A, linearizing the stem-loop structure (Fig. 1, process II). The released sequence on the stem-loop structure similarly binds to the unpaired tail of probe B, releasing a sequence identical to the initiator sequence that can also bind to probe A (Fig. 1, process III). Thus, the fluorescence-labeled amplifier probes A and B accumulate around the target sites in a form of elongated double-stranded DNA with a strong fluorescence signal (Fig. 1, process IV). Yamaguchi et al. (2015b) first applied HCR-FISH on environmental microbes, validating it by using it on active sludge samples. Nikolakakis et al. (2015) used HCR-FISH to observe the migration of syntrophic microbes on squids. Recently, Imachi et al. (2020) successfully enriched strains of Asgard archaea and documented their shapes using HCR-FISH. There are several advantages of using HCR-FISH compared to CARD-FISH. Firstly, the probes in HCR-FISH are much smaller, and so can more easily penetrate the cells (Amann and Fuchs 2008). Secondly, the fluorophores are labeled on amplifier probes rather than initiator probes, thus the initiator probes can easily be changed with demand with only a small additional cost of time and expense. Thirdly, not using H_2_O_2_ in the HCR-FISH protocol better preserves the target RNA in the sample (Massie et al. 1972). Fourthly, the HCR-FISH protocol is less time-consuming.

While the signal intensity of FISH can be improved by the above methods, the specificity of FISH can be influenced by additional factors, especially in sediment samples. Firstly, if the hybridization condition is not stringent enough, the probe may bind to non-targeted RNAs with partially complementary sequences. If this occurs, cells without target RNA may also present a probe signal. Secondly, although base-pair matching does not occur, there is still a chance that the probe will be adsorbed by abiotic particles (Amalfitano and Fazi 2008; Daims and Wagner 2007). This would be more likely in HCR-FISH as more DNA probes are involved, increasing the possibility of DNA adsorption by abiotic particles. These kinds of problems prevent the application of HCR-FISH to sediment and soil samples. Indeed, one attempt to apply HCR-FISH to sediments was unsuccessful due to strong false-positive signals (Buongiorno et al. 2017). In this study, we first validated and optimized HCR-FISH on pure cultures of the bacterium *Escherichia coli* and the archaeon *Methanococcoides methylutens*. We found that the concentration of the initiator probe in the hybridization buffer needed to be increased to 10 μmol/L and two sets of HCR sequences outcompeted others. Then, we tried to reduce the false positive rate of HCR-FISH on sediment samples. Modifications regarding pretreatment methods and hybridization reagents were validated for sediment samples. We also developed an image-processing method that could improve the performance of DAPI counter staining. Combining these methods, we successfully visualized microbes in sediment with HCR-FISH, demonstrating HCR-FISH as a promising method for use in environmental microbial community research.

## Results and discussion

### Verification of HCR-FISH on pure-cultured microbes

#### Modification of the HCR-FISH protocol

**Fig. 2:**
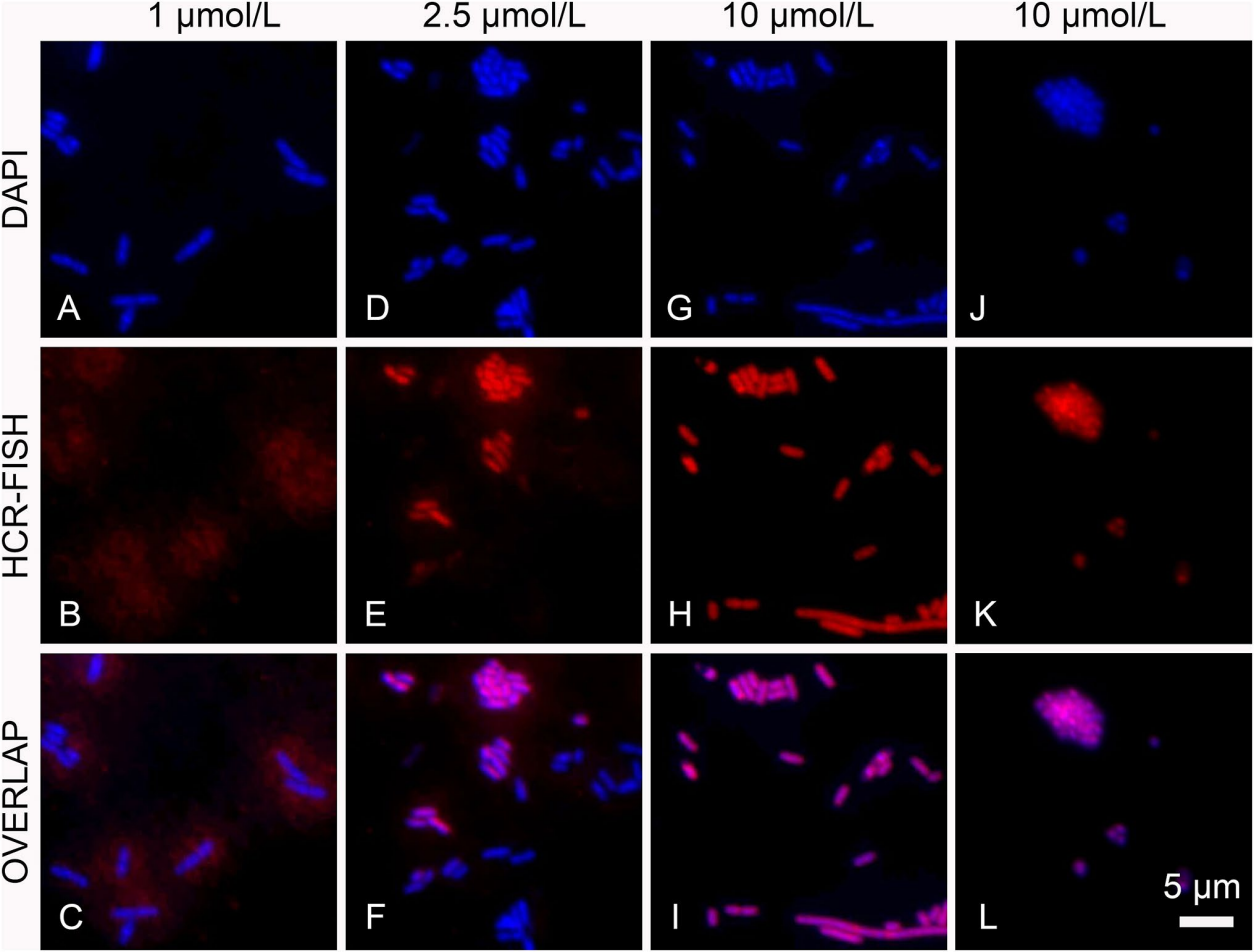
HCR-FISH on pure cultured microbes. *E. coli* was labeled with EUB338 (**a**–**i**) and *M. methylutens* was labeled with ARCH915 (**j**–**l**), both with HCR probe set S1. The concentrations of initiator probe in hybridization solution are 1 μmol/L (panel **a**–**c**), 2.5 μmol/L (panel **d**–**f**), and 10 μmol/L (panel **g**–**l**), respectively. The micrographs depict DAPI stain (left) and those depict HCR-FISH signals (middle) are overlapped on the right

To verify the protocol, HCR-FISH was first tested on the model bacterium *E. coli,* targeted by the universal bacterial probe EUB338, following the procedure proposed by Yamaguchi et al. (2015a, b). In the original protocol, the concentration of initiator probe was 1 μmol/L, similar to that used in the traditional FISH. In these conditions, the signal of HCR-FISH on *E. coli* was too unclear to identify the exact location and shape of individual cells (Fig. 2a–c). The cell signal became more intensive and clearer when the probe concentration was increased from the original 1 μmol/L to 2.5 or 10 μmol/L (Fig. 2d–i). Other modifications of the fixation procedure and hybridization state, including the temperature, formamide concentration and moisture level, etc., did not show much improvement when the concentration of the initiator probe remained at 1 μmol/L (data not shown). Therefore, the concentration of initiator probe in the hybridization buffer was set to 10 μmol/L for all further experiments. The average fluorescence signal intensity of HCR-FISH on *E. coli* (8.85 ± 1.3 arbitrary unit, A.U.) was ~ 5 times that of traditional FISH (1.61 ± 0.58 A.U.) and there were no obvious differences between the cell counting results on *E. coli* by HCR-FISH ((6.74 ± 2.14) × 10^8^ cells/ml) and by SYBR Green I staining ((7.56 ± 3.25) × 10^8^ cells/ml) (Student's *t*-test, *n*1 = *n*2 = 3, *P* > 0.1). The modified protocol also worked well on pure cultures of the archaeon *Methanococcoides methylutens* (Fig. 2j–l). While several groups have independently conducted the HCR-FISH protocols of Yamaguchi et al. (2015a, b), they did not arrive at a unified conclusion on the suitability of this protocol (Buongiorno et al. 2017; Francis et al. 2019; Grieb et al. 2020; Matsubayashi et al. 2017; Royet et al. 2018). Therefore, it is likely that some experimental conditions suggested in the Yamaguchi et al. (2015a, b) protocols are only marginally suitable and may need further optimization to achieve robust performance. Here, it is proposed that increasing the concentration of the initiator probe in the hybridization buffer improves the original protocol. In this study, the method was validated by two relatively independent labs.

#### Test of HCR probe sets

Observing phylogenetically distinct microbes simultaneously allows more application of HCR-FISH. In order to achieve this goal, at least two sets of HCR sequences are required and they should meet the following criteria: (a) these sequences should be orthogonal, meaning that they won’t hybridize with each other; and (b) using identical protocols, they should give an acceptable performance under the same working conditions, i.e., they will give a strong signal with the targeted microbes and weak signals with the untargeted materials. Based on previous studies (Choi et al. 2014; Yamaguchi et al. 2015b), five orthogonal HCR sequence sets named S1/S2/S3/L1/L2 (Table 1) were chosen and tested on *E. coli*. Those that worked well under the modified protocol were selected for further experiments. Among them, S1, S2, and S3 were shorter in length, with initiator sequences consisting of 26 nucleotides, while those of L1 and L2 had 36 nucleotides each. Each initiator sequence was fused with bacterial universal probe EUB338. It was revealed that the shorter probe sets, S1 and S2, gave stronger fluorescence signals, while L1 and L2 emitted only vague and unclear signals, similar to the results of the S1 probe using a probe concentration of 1 μmol/L (Supplementary Fig. S1a, b). It is possible that the shorter length gave S1 and S2 a better opportunity to enter the cells and interact with their targeted RNA, while a longer length made L1 and L2 more likely to be influenced by steric factors. Based on these results, experiments with L1 and L2 were discontinued. The S3 probe set resulted in many nonspecific binding signals outside the cells (Supplementary Fig. S1c), consistent with a previous study (Buongiorno et al. 2017). Comparatively, the presence of S1 and S2 signals were well limited in the neighborhood of DAPI signal, showing high specificity (Fig. 2g–i, Supplementary Fig. S1d). It’s possible that the sequence of S3 itself tended to trigger nonspecific binding. At last, probe S1 and S2 were selected for further study because of their high signal intensity, clearness, and specificity.

**Table 1:** Probes used in this study

Probe name	Probe sequence (5′-3′)	Reference
S1a	TCTAGTCGTTGATGCTTTGTATTCGGCGACAGATAACCGAATACAAAGCATC	Choi et al. (2010)
S1b	CCGAATACAAAGCATCAACGACTAGAGATGCTTTGTATTCGGTTATCTGTCG	Choi et al. (2010)
S2a	CATAGGGTTCGGATTCTTAGGGCGTAGCAGCATCAATACGCCCTAAGAATCC	Choi et al. (2010)
S2b	TACGCCCTAAGAATCCGAACCCTATGGGATTCTTAGGGCGTATTGATGCTGC	Choi et al. (2010)
S3a	ATGAAGGACGGACTACTGATAACTGGGACTTCCATACCAGTTATCAGTAGTC	Choi et al. (2010)
S3b	CCAGTTATCAGTAGTCCGTCCTTCATGACTACTGATAACTGGTATGGAAGTC	Choi et al. (2010)
L1a	GAAGCGAATATGGTGAGAGTTGGAGGTAGGTTGAGGCACATTTACAGACCTCAACCTACCTCCAACTCTCAC	Choi et al. (2014)
L1b	CCTCAACCTACCTCCAACTCTCACCATATTCGCTTCGTGAGAGTTGGAGGTAGGTTGAGGTCTGTAAATGTG	Choi et al. (2014)
L2a	CGGGTTAAAGTTGAGTGGAGATATAGAGGCAGGGACAAAGTCTAATCCGTCCCTGCCTCTATATCTCCACTC	Choi et al. (2014)
L2b	GTCCCTGCCTCTATATCTCCACTCAACTTTAACCCGGAGTGGAGATATAGAGGCAGGGACGGATTAGACTTT	Choi et al. (2014)
ARCH915-S1	CCGAATACAAAGCATCAACGACTAGAAAAAAGTGCTCCCCCGCCAATTCCT	Yamaguchi et al. (2015a, b)
ARCH915-S3	CCAGTTATCAGTAGTCCGTCCTTCATTTTTTGTGCTCCCCCGCCAATTCCT	This study
EUB338-S1	CCGAATACAAAGCATCAACGACTAGAAAAAAGCTGCCTCCCGTAGGAGT	Yamaguchi et al. (2015a, b)
EUB338-S2	TACGCCCTAAGAATCCGAACCCTATGAAAAAGCTGCCTCCCGTAGGAGT	This study
EUB338-S3	CCAGTTATCAGTAGTCCGTCCTTCATTTTTTGCTGCCTCCCGTAGGAGT	This study
EUB338-L1	GTCCCTGCCTCTATATCTCCACTCAACTTTAACCCGAAAAAAGCTGCCTCCCGTAGGAGT	This study
EUB338-L2	CCTCAACCTACCTCCAACTCTCACCATATTCGCTTCAAAAAAGCTGCCTCCCGTAGGAGT	This study
ANME-1-350-S1	CCGAATACAAAGCATCAACGACTAGAAAAAAAGTTTTTCGCGCCTGATGC	This study
SEEP2-658-S2	TACGCCCTAAGAATCCGAACCCTATGAAAAATCCACTTCCCTCTCCGGT	This study

### Decreasing the negative effects of abiotic particles on HCR-FISH

Through sorption of probes and fluorophores, abiotic particles in sediment significantly interfere with the performance of traditional FISH and HCR-FISH. Furthermore, large quantities of abiotic particles can cover the microbes during cell embedding (on a filter membrane), causing problems for observation. These negative effects can be alleviated by reducing either the number of abiotic particles or the adsorption of abiotic particles on the probes. To reduce these negative effects several methods for improving the performance of FISH on sediments were tested.

#### Removal of abiotic particles

Sediment particles and cells can be easily separated due to their large differences in density, but first, the cells need be detached from the abiotic particles by physical and/or chemical means to avoid cell loss during separation. Several detachment methods were tested on paraformaldehyde-treated sediment samples. Method A uses a buffer with detergent to wash the cells off the abiotic particles, while method B uses an ultrasonic probe (Kallmeyer et al. 2008). Both methods were evaluated by counting the total cell number in their supernatant after an hour of precipitation. The cell counting results obtained using methods A, B and that result of the control group were 5.70 ± 1.89, 3.20 ± 0.52, 2.80 ± 0.25 per nanoliter, respectively. Compared with the untreated sample, more cells were obtained in the supernatant through method A (Student's *t*-test, *n*1 = *n*2 = 5, *P* < 0.01), whereas method B had no obvious effect (Student's *t*-test, *n*1 = *n*2 = 5, *P* > 0.1).

For the separation step, a multi-layer density gradient centrifugation method was tested on samples treated with method A. Multiple density layers were thought to help avoid the co-precipitation of microbes with abiotic particles in the turbulent flow caused by falling of abiotic particles (Morono et al. 2013). Compared with the group that only stood still for one hour as separation, density gradient centrifugation yielded higher cell numbers, and less cells remained in the pellet (Table 2). Thus, multi-layer density gradient centrifugation was adopted in the protocol for cell separation in further experiments.

**Table 2:** Performance of different extraction methods on sediment samples from South China Sea

Cell number (10^5^cells/ml)	DG	Control
Supernatant	18.55 ± 2.94	4.30 ± 1.54
Pellet	4.51 ± 1.07	8.74 ± 1.07

*DG* density gradient centrifugation method. The number of cells in the supernatant and pallet after centrifugation was counted separately. See Supplementary Table S1 for more information

#### Reducing the sorption of initiator probe by abiotic particles

To reduce false-positive signals resulting from the nonspecific binding of abiotic particles on probes, three types of hybridization buffer (A, B, C) were tested (Table 3). Buffer A is a widely used formula for FISH on environmental samples (Yamaguchi et al. 2015b). Buffer B is mostly used with eukaryote samples, with several traditional blocking reagents amended (Choi et al. 2014). Buffer C contains a high concentration of EDTA (Morono et al. 2020). Our experiments demonstrated that Buffer B did not work well on sediment samples, as nonspecific binding remained significant. The performance of Buffer C outcompeted that of Buffer A in decreasing the nonspecific binding. Buffer C was thus selected for further experiments.

**Table 3:** Composition of hybridization buffer tested on sediment samples

Main ingredients	Hybridization buffer A	Hybridization buffer B	Hybridization buffer C
Tris–HCl	20 mmol/L	–	20 mmol/L
EDTA	0	–	250 mmol/L
SDS	0.01% (w/v)	–	0.01% (w/v)
NaCl	0.9 mol/L	–	–
Dextran sulfate	10% (w/v)	10% (w/v)	–
5× Sodium chloride sodium citrate	–	5×	–
Citric acid	–	9 mmol/L	–
1× Denhardt’ solution	–	1×	–
Tween 20	–	0.1% (v/v)	–
Heparin	-	50 μg/ml	–
Formamide	X% (v/v)	X% (v/v)	X% (v/v)
reference	Yamaguchi et al. (2015a)	Choi et al. (2014)	Morono et al. (2020)

### Imaging and image processing to reduce false counterstaining signal

In FISH experiments, DNA counterstaining is often applied to locate cells. A widely used counterstaining dye is DAPI, which shows specificity on double-stranded DNA. However, sediment particles, with complex compositions and structures, inevitably absorb DAPI molecules and emit a background fluorescence signal. In practice, using one band-pass DAPI filter set, it is difficult to differentiate cells and abiotic particles based solely on their shape and intensity. Similarly, the DNA dye SYBR Green is also absorbed by abiotic particles, although it was found that the emission spectrum of SYBR Green goes through a redshift when it binds to abiotic particles rather than DNA (Sunamura et al. 2003). By using a band-pass filter 490/20 nm (center wavelength/bandwidth) for excitation and 528/38 nm and 617/73 nm filters for detection, abiotic particles could easily to be ruled out (Morono et al. 2009). However, SYBR Green produces signals under several fluorescence channels and thus may not be compatible with FISH.

**Fig. 3:**
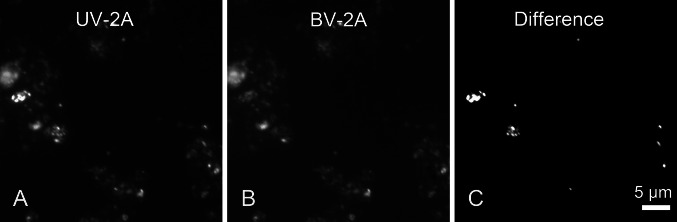
Image processing excludes false positive signal of DAPI stain. Image taken from UV-2A channel (**a**) subtracts that taken from BV-2A channel (**b**) to produce a new image with enhanced signal of cells and decaying signal of abiotic particles (**c**)

In the hope that DAPI might have similar properties to SYBR Green, several filter sets were tested. Our results showed that DAPI-DNA complex only emitted a bright signal under light filter UV-2A (excitation filter 355/50 nm, dichroic mirror 400 nm, barrier filter 410 nm), while the DAPI-abiotic-particle complex emitted signals both under UV-2A and BV-2A (excitation filter 420/40 nm, dichroic mirror 455 nm, barrier filter 460 nm). Based on this property, images under UV-2A (Fig. 3a) and BV-2A (Fig. 3b) were acquired, separately, then the latter images were subtracted from the former. This process was able to dramatically decrease the signal of abiotic particles, while still retaining the signal of cells (Fig. 3c). The spectrum shift of the DAPI signal on abiotic particles may be related to the fact that DAPI has two binding modes to DNA, each of which has an independent fluorescence spectrum and fluorescence quantum yield (Manzini et al. 1983). According to previous studies, mineral particles in the sediment sample can absorb debris DNA to become DAPI-bindable (Krsek and Wellington 1999). It is possible that DAPI bindings to debris DNA and genomic DNA have different preferences for binding modes. The different ratio of each type of binding modes on debris DNA and genomic DNA may cause the shift of spectrum.

### Detecting microbial cells in sediment samples

**Fig. 4:**
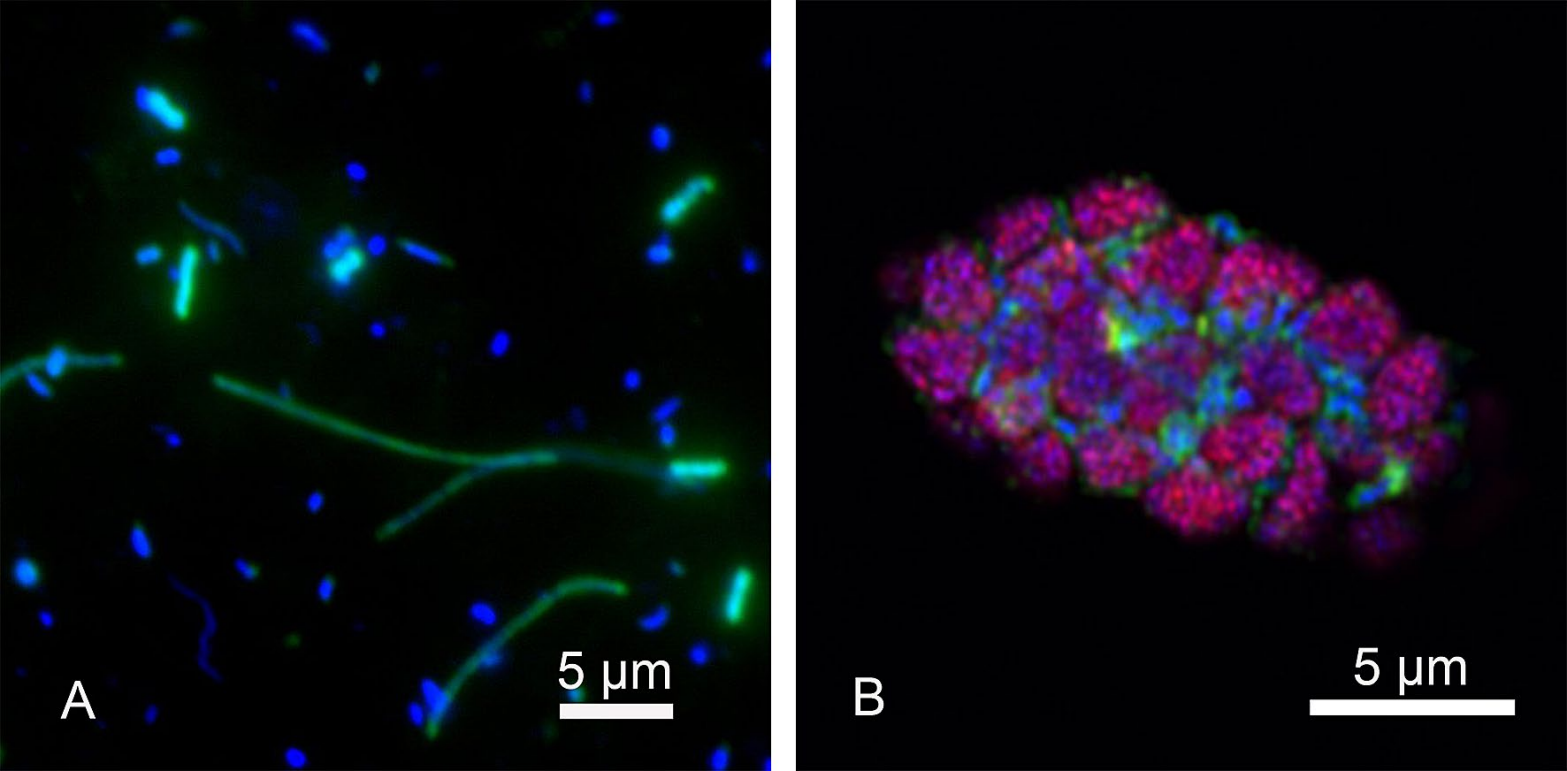
HCR-FISH on two environmental samples. **a** HCR-FISH on sediment sample from South China Sea. Bacteria were labeled by EUB338-S1. Scale bar, 5 μm. **b** HCR-FISH on an enrichment sample of anaerobic methanotrophic archaea (ANME) consortia. SRB and ANME were labeled by species-specific probe SEEP2-658-S1 and ANME-1-350-S2, respectively

**Fig. 5:**
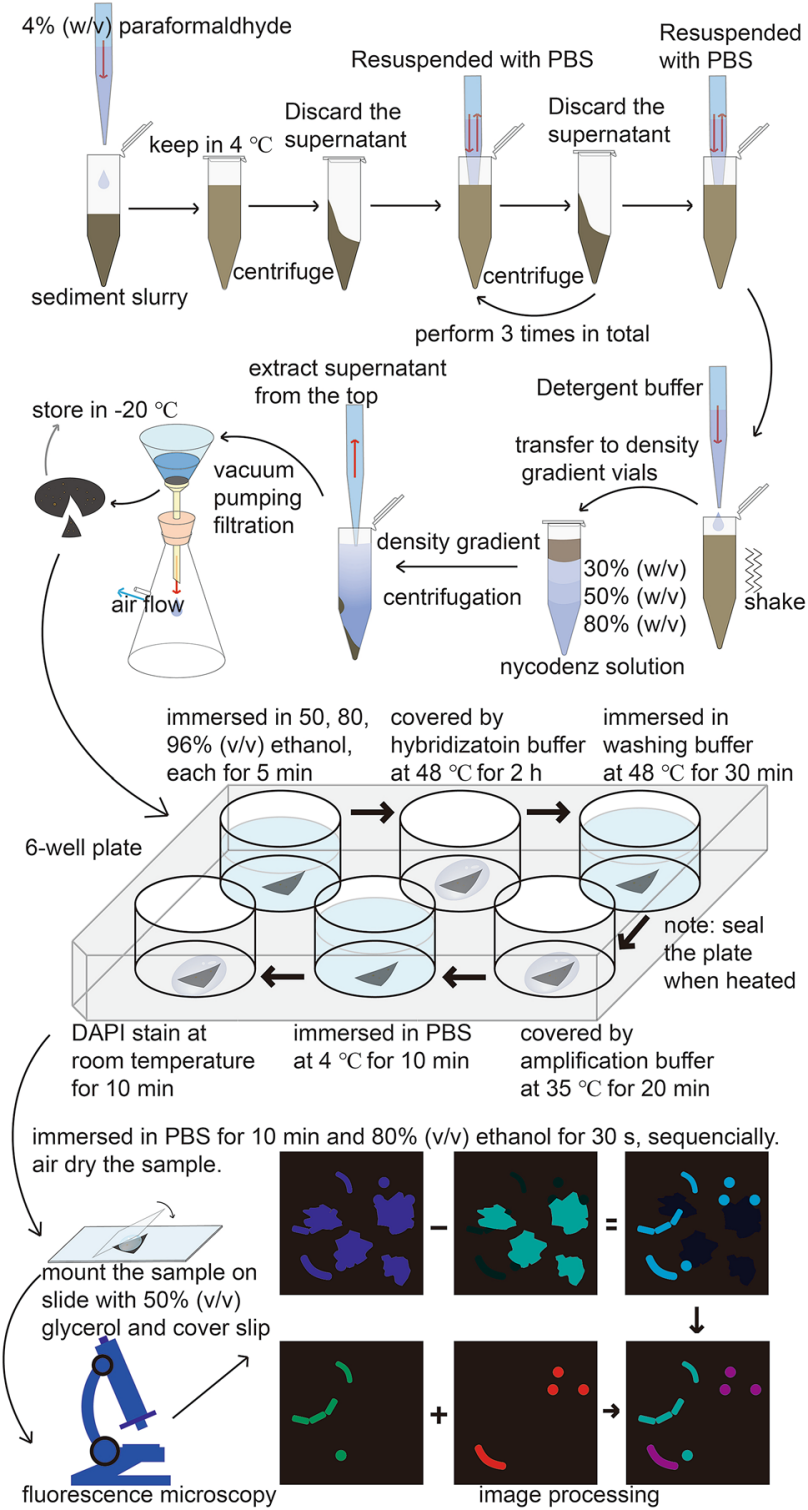
Cartoon showing the complete protocol of HCR-FISH on sediment sample. The sediment slurry is fixed by paraformaldehyde and then washed with PBS. Cells are detached through shaking in the detergent buffer and extracted through density gradient centrifugation. 0.22 μm-pore-size polycarbonate membrane is used for capturing cells. A piece of membrane is placed in the container like a 6-well plate. After dehydration with series ethanol solution, the air-dry membrane is covered by hybridization buffer and amplification buffer sequentially, each followed a washing step. Then the sample is stained with DAPI and mounted on the slide for microscopy. The image taken under BV-2A channel was subtracted from that under UV-2A channel for cell recognition and counting. The result could be overlaid with FISH probe signals for further analysis

After improving the performance of HCR-FISH on sediment samples, an optimized protocol was proposed (Supplementary Table S1 and Fig. 5). A sediment sample from the South China Sea and an anaerobic methanotroph enrichment from the Guaymas Basin sediment (Krukenberg et al. 2018) were further tested using the optimized protocol as shown in Fig. 5. After HCR-FISH, samples were counterstained with DAPI and microscopically examined. A post-imaging process was necessary to discriminate cells from fluorescent abiotic particles. As shown in Fig. 4, microbial cells could be clearly visualized by HCR-FISH, both on sediments from South China Sea and the enrichment slurry sample. For the South China Sea sample, traditional FISH was also applied for comparison. The average fluorescence intensity of the signals from bacteria were 10.9 ± 2.1 and 1.79 ± 0.60 A.U. through HCR-FISH and traditional FISH, respectively. There is thus a ~ 6× elevation of signal intensity using the HCR process. It was also noteworthy that, although multi-layer density gradient centrifugation was applied to this sample, many abiotic particles still remained. This indicates that many abiotic particles have a similar density to the cells. During cell embedding (on a filter membrane), the accumulation of these types of abiotic particles will also cover the cell, making the improvement by density gradient centrifugation less than expected.

### Potential of HCR-FISH

In addition to the application to marine sediment samples, our modified protocol may also be directly applied to concentrated seawater samples, which share the same features of low cellular rRNA content. The 6× signal enhancement ensures a better sensitivity of HCR-FISH than that of traditional FISH. The better penetration performance makes HCR-FISH more competitive on unknown samples than CARD-FISH, whose permeabilization process is empirical and sample dependent (Amann and Fuchs 2008). Meanwhile, the DAPI-related image processing method could also be integrated into other fluorescence imaging experiments, including CARD-FISH and auto-fluorescence observation, to help identify cells from false-positive signals.

In addition to detecting microbes, single-cell level detection of mRNA can be more appealing to researchers studying unculturable microbes. For example, study of *mcr*A transcripts in methanogens using a two-step CARD-FISH process (i.e., two-pass Tyramide Signal Amplification FISH) shows the possibility to detect mRNA in microbes by FISH (Kubota et al. 2006). However, this method has not been widely used, probably because of its complexity. Some studies have used single-molecular FISH (smFISH), a method that involves a set of more than 40 probes targeting different regions on the sequence of mRNA (Sepulveda et al. 2016; Skinner et al. 2013; So et al. 2011; Taniguchi et al. 2010; Yang et al. 2019), to locate mRNA molecules inside *E. coli*. Furthermore, genes could also be visualized by signal-enhanced FISH like virusFISH (Castillo et al. 2020) or geneFISH (Barrero-Canosa et al. 2017; Moraru et al. 2010), which combines the strategies of dense fluorescent labeling of single probe and multiple probes. Considering the success of CARD-FISH and smFISH on in situ detection of single molecules in microbes, it would also be expected that HCR-FISH would be an effective single-molecule detection tool based on its efficient signal amplification and relatively easy protocol.

## Conclusions

The traditional FISH, with a single probe, was not sensitive enough to explore tiny and/or less active microbes in many natural environments, such as marine sediments (Ishii et al. 2004) and open ocean seawater (Morris et al. 2002). Several new technologies have been developed to deal with this problem. In this study, we have demonstrated that HCR-FISH, one of these technologies, can significantly improve the labeling of microbes in sediment samples. The overall performance of the original HCR-FISH on sediment samples could be improved by applying the following modifications: 1) The signal intensity and resolution of HCR-FISH can be optimized through changing the concentration of initiator probes and types of HCR sequences; 2) The negative effects of abiotic particles in HCR-FISH of sediment samples can be reduced by employing sample pretreatment and optimized hybridization buffer; 3) The counterstaining signal of cells could be distinguished from the background fluorescence of abiotic particles by performing an image processing method to emphasize the cellular DAPI signal. Combining these efforts, we developed an optimized HCR-FISH protocol for sediment samples (Supplementary Table S1 and Fig. 5).

## Materials and methods

### Sample preparation

*E. coli* and *M. methylutens* were selected as representatives of bacteria and archaea, respectively. *E. coli* DH5α was cultured in Luria–Bertani (LB) broth at 37 ℃ under 200 rpm shaking. *M. methylutens* DSM 16625 was obtained from the German Collection of Microorganisms and Cell Cultures (DSMZ, Braunschweig, Germany) and cultured according to DSMZ protocols. Cells were fixed in 4% (v/v) paraformaldehyde with phosphate-buffered saline (PBS; 136 mmol/L NaCl, 2.6 mmol/L KCl, 8 mmol/L Na_2_HPO_4_, and 2 mmol/L KH_2_PO_4_ [pH 7.2]) for 6 h at 4 ℃, washed twice by PBS, and preserved in PBS/ethanol 1:1 (v/v) mixture at −20 °C.

Sediment samples were collected from the South China Sea and stored at −80 °C before proceeding to HCR-FISH. The same fixation and preservation procedures, as described above and in Supplementary Table S1, were applied on these sediment samples.

An enrichment sample of anaerobic methanotroph was transferred from a long-term enrichment incubator at the Max Planck Institute for Marine Microbiology, Germany. The enrichment was transferred and refreshed according to the reference (Krukenberg et al. 2018). The same fixation and preservation procedures, as described above and in Supplementary Table S1, were also applied.

### Cell detachment

Cells were detached from fixed samples using different methods. For method A, eight volumes of sediment were mixed with one volume of detergent buffer (100 mmol/L EDTA, 100 mmol/L sodium pyrophosphate, 1% (v/v) Tween 80) and vortexed for one hour at level 5 (Vortex-Genie 2, Scientific Industries). For method B, samples were placed in an ice-water mixture 2 cm away from an ultrasonic probe (Model 50 Sonic Dismembrator, Thermofisher). A 30-s sonication with 50% maximum power was performed three times, each followed by a resting period of 30 s. To evaluate their performance, detached samples were rested for an hour to precipitate the particles, and the supernatant was used for the cell counts. A control group was also set with fixed sediment sample shaken, rested, and counted as for the other groups.

### Cell extraction

Cells were extracted from the detergent buffer detached samples with density gradient centrifugation. Samples were centrifuged at 1500 *g* for 30 min through multiple density layers consisting of 30%, 50%, and 80% (w/v) Nycodenz (Axis-Shield, Norway), respectively. All the transparent liquid was carefully transferred to a clean vial to avoid the disturbance on the precipitate. Then, the supernatant was diluted 4 times by PBS and centrifuged at 14,500*g* for 15 min to collect the cells. The precipitated cells were resuspended in PBS for further processing. To compare, parts of the detached samples were allowed to stand for an hour to precipitate the abiotic particles and suspend cells. Their supernatants were collected and stored in PBS for further processing.

### Cell counting

To capture the cells, pretreated samples were first diluted in 10 ml PBS and then filtered onto 0.22 μm pore size membranes (GTBP02500, Isopore, Merck Millipore). Then, the membrane was dipped in 50, 80, and 96% (v/v) ethanol for dehydration, each step was for 5 min (Yamaguchi et al. 2015a). SYBR Green I (Solarbio, China) was diluted in ultrapure water by 100 times to provide the working solution. The working solution was then applied to the membranes containing the samples for 10 min at room temperature. Then, the membranes were placed on the top of the delicate task wipes (Kimtech Science, USA). The remaining working solution would be absorbed by the delicate task wipes. For each filter membrane, 16 fields of view were selected and counted under the microscope. Three filter membranes were analyzed per sample.

### HCR-FISH

The HCR-FISH protocol was modified based on that of Yamaguchi et al. (2015a). Cells were captured on membranes as described in “Cell counting” section. The membrane was cut into eight pieces, each of which was enough for the following experiment. The unused pieces were stored in −20 °C for future usage. 40 μl of hybridization buffer with 10 μmol/L initiator probes was added to the membrane. The concentration of formamide in the hybridization buffer depends on the sequence of the probe. As tested here, 25% (v/v) formamide was acceptable for simultaneous detection of microbes using probe EUB338, ARCH915, ANME-1-350 and SEEP2-658. The sample was incubated at 46 °C for 2 h in humidified conditions. The humidity requirement can be achieved by sealing the sample in a small container and/or placing a wet tissue beside the sample. After incubation, the membrane was washed in 10 ml washing buffer [20 mmol/L Tris–HCl, 0.01% (w/v) SDS, 0.056–0.225 mol/L NaCl] and incubated at 48 ℃ for 30 min to remove excessive initiator probes. The concentration of NaCl in the washing buffer depends on the concentration of formamide in the hybridization buffer. Based on the Pernthaler et al*.* (2001), 0.056 mol/L NaCl was used for 40% (v/v) formamide, 0.159 mol/L NaCl for 25% (v/v) formamide and 0.225 mol/L NaCl for 20% (v/v) formamide. A complete table of concentration pairs can be found in Pernthaler et al*.* (2001). All the following steps should be done in dark to avoid quenching of the fluorophore. During washing, each amplifier probe was dissolved in amplification buffer [0.9 mol/L NaCl, 0.05 mol/L Na_2_HPO_4_, 0.01% (w/v) SDS] and incubated stepwise at 95 °C for 90 s and 25 °C for 30 min, for initialization. Next, these amplifier probes were mixed and the final concentration of each probe was 2.5 μmol/L. 30 min later, the washing buffer was removed and 40 μl amplifier probes mixed with amplification buffer was added to the membrane. The membrane was then incubated at 35 °C for 20 min under humidified conditions. Sequentially, 10 ml 4 °C PBS was applied to the membrane on ice to remove excessive fluorescent probes. The membrane was washed in ultrapure water and then in 96% (v/v) ethanol each for one minute on ice and air-dried.

For counterstaining, 20 μl of 10 ng/ml DAPI was applied to the samples for at least 10 min. Samples were washed by PBS for 10 min and dehydrated by 80% (v/v) ethanol.

### Traditional FISH

Samples underwent the same treatment as those in the HCR-FISH protocol until the hybridization step. 40 μl of hybridization buffer with 10 μmol/L of fluorophore-labeled probes was added to the membrane. The sample was incubated and washed following the same procedure as for HCR-FISH. Then, the membrane was directly washed in water and 96% (v/v) ethanol, each for one minute, on ice and air dried. The counterstaining step remained the same.

### Imaging and image processing

The imaging experiment was carried out by epifluorescence microscope (Nikon ECLIPSE 90i, Tokyo, Japan), coupled with an illuminator (Nikon INTENSILIGHT C-HGFIE, Tokyo, Japan) and a CCD camera (CoolSNAP HQ2, Photometrics, USA). Nikon plan-apochromat 100× oil objective lens was used for imaging. The excitation filter, dichroic filter, and barrier filter of light filter cubes are summarized in Table 4. The camera exposure time varied between 50 and 800 ms depending on the type of fluorophore and observed samples. Images of anaerobic methanotrophs were collected with a Zeiss LSM 880 microscope (Carl Zeiss, Germany) equipped with a plan-apochromat 63× oil objective lens and Airyscan super-resolution system for better resolution. 405, 561, and 633 nm lasers were used for excitation of DAPI, Alexa Fluor 555, Alexa Fluor 647, respectively.

**Table 4:** Filter information of light filter cubes in microscope for this study

	Excitation filter	Dichroic filter	Barrier filter
UV-2A long-pass filter set	355/50 nm^a^	400 nm	410 nm
BV-2A long-pass filter set	420/40 nm	455 nm	460 nm
DAPI band-pass filter set	375/28 nm	415 nm	460/60 nm
FITC band-pass filter set^b^	480/30 nm	505 nm	535/45 nm
Texas Red band-pass filter set^c^	560/40 nm	595 nm	630/60 nm

^a^Central wavelength/bandwidth, the same hereinafter

^b^Used for fluorophore Alexa 488

^c^Used for fluorophore Alexa 594

The channel subtraction was performed using the Image Calculator function integrated in Fiji (a "batteries-included" distribution of ImageJ 1.53c) software released on 2017 May 30 (Schindelin et al. 2015). To balance the background differences between channels, the images were treated under the following rules: define the gray value that most pixels possessed for images from UV-2A and BV-2A channels as I_U_ and I_B_, respectively. Both *I*_U_ and *I*_B_ represent the low-intensity background in the corresponding image channels. Hence, pixels with a grey level of *I*_U_ in UV-2A channel and those with grey level of *I*_B_ in BV-2A channel should occupy the same positions in the image. Then, the grey values of these pixels should become zero by channel subtraction to precisely eliminate the background signal. This could be achieved by multiplying the grey value of each pixel on the image from BV-2A by a factor *α* = *I*_U_/*I*_B_. Practically, *α* ≈ 1/8 for our instruments, and thus the goal could also be achieved by adjusting the exposure time or exciting light intensity for an image from BV-2A to 1/8 of that of the image from UV-2A. The factor α may vary with different light sources, but not with samples.

The analysis of cellular fluorescence intensity was also performed on Fiji. To identify the region of positive signal for each channel, two binary images were created from the channel-subtracted DAPI image and (HCR-)FISH image, separately. The thresholds for producing these binary images were decided based on the built-in automatic algorithm “Moments”. The intersection of the two binary images was used as the cellular mask for intensity calculation of true cells. To measure the cellular fluorescence intensity, we first designated the (HCR-)FISH image to be calculated in the “Set Measurements…” window. Then the window of the cellular mask image was activated and the “Analyze Particles” function was run. The “target size” parameter of the function was set to be 0.5–100 mm^2^. With this pipeline, the Fiji software would first mark the cell regions using the cellular mask image, and then calculate the average grey value of each region on the (HCR-)FISH image. For each experiment group, at least 200 cells were taken into account.

## Supplementary Information

Below is the link to the electronic supplementary material.

Supplementary file1 (DOCX 124 KB)

Supplementary file2 (DOCX 24 KB)
