# Supplementary material for: Optimizing the hybridization chain reaction-fluorescence in situ hybridization (HCR-FISH) protocol for detection of microbes in sediments
Source: Mar Life Sci Technol. 2021 Apr 30;3(4):529–41. doi: 10.1007/s42995-021-00098-8 (PMC10077247; doi:10.1007/s42995-021-00098-8)
Supplement: Supplementary file 2 — Supplementary file2 (DOCX 24 KB) [file 42995_2021_98_MOESM2_ESM.docx]

published on **Marine Life Science & Technology**

**Optimizing hybridization chain reaction with fluorescence *in situ* hybridization (HCR-FISH) protocol for detection of microbes in sediments**

Zeyu Jia^a^, Yijing Dong^b^, Heng Xu^b,c†^, Fengping Wang^a,d*^

^a^ State Key Laboratory of Microbial Metabolism, Joint International Research Laboratory of Metabolic & Developmental Sciences, School of Life Sciences and Biotechnology, Shanghai Jiao Tong University, Shanghai 200240, China

^b^ School of Physics and Astronomy, Shanghai Jiao Tong University, Shanghai 200240, China

^c^ Institute of Natural Science, Shanghai Jiao Tong University, Shanghai 200240, China

^d^ School of Oceanography, Shanghai Jiao Tong University, Shanghai 200240, China

^†^Co-corresponding author: E-mail address: Heng_Xu@sjtu.edu.cn

^*^Corresponding author: E-mail address: fengpingw@sjtu.edu.cn

**Supplementary Table S1** Optimized HCR-FISH protocol on sediment samples. Sentences in italics indicate major modifications there, on the basis of original HCR-FISH protocol.

| Fixation | 1. Immerse the sample in 4% (v/v) paraformaldehyde in centrifuge tube and mix them by shaking. Then keep the tube at 4 ℃ for 6 h. 2. Centrifuge the tube at 12000× g for 10 min, discard the supernatant. Resuspend the pellet with PBS. Repeat this process for a total of 3 times. This process could wash off the excessive formaldehyde. the sample is ready for cell detachment, or be stored after step 3. 3. Centrifuge the tube at 12000× g for 10 min. discard the supernatant. Resuspend the pellet with PBS and equal volume of dehydrated ethanol. the sample could be preserved at -20 ℃ for long time. |
| --- | --- |
| *Cell detachment* | 1. *Mix 8 volume of fixed sediment sample with 1 volume of detergent buffer and vortex for 1 h.* |
| *Cell extraction* | 1. *Prepare the density gradient vial by first adding light Nycodenz solution to the EP tube and then inject heavier Nycodenz solution from the bottom of the lighter layer slowly. Each layer consists of 0.5 ml liquid.* 2. *Transfer 0.3 ml pretreated sample to the top of density gradient vial. Each sample may be transferred to several vials to acquire large cell quantity in total.* 3. *Centrifuge the vials at 1500*× *g for 30 min.* 4. *Carefully transfer the upper layer to clean tubes from the interface, this could ensure that the floating cells are captured.* 5. *Retrieve the cells by vacuum filtration on 0.22 μm-pore-size polycarbonate membrane.* |
| Hybridization | 1. 1/8-1/4 piece of membrane were used to a round of HCR-FISH. Place the piece in a container. 2. *40 μl Hybridization buffer C with 10 μmol/L initiator probe was added on the membrane.* 3. Seal the container airtightly. Place the container in a force-convection incubator at 46 ℃ for 2 h. 4. Wash the membrane by pouring 10 ml wash buffer into the container, place it at 48 ℃ for 30 min. 5. Pick out the membrane and wipe the liquid with tissue from the back of membrane. Place it in a clean container. |
| Amplification | 1. During the washing step, preheat each amplifier probes in amplification buffer at 95 ℃ for 1.5 min, and cool it down at 25 ℃ for 30 min. 2. Mix the probes to a final concentration of 2.5 μmol/L per probe. 3. Add 20 μl of this mixture on the sample, keep the container in 35 ℃ for 20 min. 4. Wash the membrane by pouring 10 ml PBS into the container, place it on ice for 10 min. 5. Pick out the membrane and wipe the liquid with tissue from the back of membrane. Place it in a clean container. |
| Counterstain | 1. Add 20 μl DAPI solution on the membrane, stain it at 25 ℃ for at least 10 min. 2. Wash off the DAPI by PBS at 25 ℃ for 10 min. 3. Dehydrate the membrane with 80% (v/v) ethanol for 1 min. air dry the membrane. The membrane is ready for mounting and microscopy. |
| *Post-imaging process* | 1. *Subtract the micrograph taken under channel BV-2A from that under channel UV-2A* |
